# POLE Score: a comprehensive profiling of programmed death 1 ligand 1 expression in pancreatic ductal adenocarcinoma

**DOI:** 10.18632/oncotarget.26705

**Published:** 2019-02-22

**Authors:** Sascha Rahn, Sandra Krüger, Ruben Mennrich, Lisa Goebel, Daniela Wesch, Hans-Heinrich Oberg, Ilka Vogel, Michael Ebsen, Christoph Röcken, Ole Helm, Susanne Sebens

**Affiliations:** ^1^ Institute for Experimental Cancer Research, Christian-Albrechts-University Kiel (CAU) and University Medical Center Schleswig-Holstein (UK-SH), Campus Kiel, Kiel, Germany; ^2^ Department of Pathology, CAU and UK-SH, Campus Kiel, Kiel, Germany; ^3^ Institute of Immunology, CAU and UK-SH, Campus Kiel, Kiel, Germany; ^4^ Department of Surgery, Community Hospital Kiel, Kiel, Germany; ^5^ Institute of Pathology, Community Hospital Kiel, Kiel, Germany

**Keywords:** PD-L1, pancreatic cancer, tumor stroma, prognostic relevance

## Abstract

Pancreatic ductal adenocarcinoma (PDAC) being characterized by a pronounced stromal compartment is commonly diagnosed at an advanced stage limiting curative treatment options. Although therapeutical targeting of immune checkpoint regulators like programmed death 1 ligand 1 (PD-L1) represent a promising approach that substantially improved survival of several highly aggressive malignancies, convincing indicators for response prediction are still lacking for PDAC which might be attributed to the insufficient characterization of PD-L1 status. Therefore, we investigated PD-L1 expression by immunohistochemistry in a well characterized cohort of 59 PDAC and 18 peritumoral tissues. Despite the histopathological homogeneity within our cohort, tumor tissues exhibited a great heterogeneity regarding PD-L1 expression. Considering distinct PD-L1 expression patterns, we established the novel POLE Score that incorporates overall PD-L1 expression (P), cellular Origin of PD-L1 (O), PD-L1 level in tumor-associated Lymph follicles (L) and Enumerated local PD-L1 distribution (E). We show that tumoral PD-L1 expression is higher compared to peritumoral areas. Furthermore, POLE Score parameters correlated with overall survival, tumor grade, Ki67 status, local proximity of tumor cells and particular stroma composition. For the first time, we demonstrate that PD-L1 is mostly expressed by stroma and rarely by tumor cells in PDAC. Moreover, our *in situ* analyses on serial tissue sections and *in vitro* data suggest that PD-L1 is prominently expressed by tumor-associated macrophages. In conclusion, POLE Score represents a comprehensive characterization of PD-L1 expression in tumor and stroma compartment and might provide the basis for improved patient stratification in future clinical trials on PD-1/PD-L1 targeting therapies in PDAC.

## INTRODUCTION

Pancreatic ductal adenocarcinoma (PDAC) is the fourth leading cause of cancer related deaths in Western countries [1]. The dismal 5-year survival rate of < 8% is mainly attributed to the lack of reliable biomarkers and screening methods for early detection of the tumor. Therefore, more than 80% of PDAC patients are diagnosed at locally advanced or metastatic stages [2]. However, despite the advances in research on PDAC development and progression, palliative chemotherapy represents the only treatment option for patients with unresectable tumors [3].

The extensive desmoplastic stroma, which constitutes almost 80% of the tumor mass, has been shown to promote aggressiveness and treatment resistance of PDAC [4]. Highlighting the role of the cellular stromal compartment, the increased presence of immunosuppressive cell populations, e.g. tumor-associated macrophages (TAMs) and regulatory T cells (T_regs_), is related to a poor prognosis [5, 6]. Hence, immunotherapeutical approaches are promising novel strategies to reactivate tumor directed immune responses. Based on the impressive response rates in several other malignancies, e.g. non-small cell lung carcinoma and melanoma, blocking of the programmed cell death protein 1 (PD-1)/programmed cell death 1 ligand 1 (PD-L1) signaling pathway currently represents one the most intensively investigated immune therapies for the treatment of PDAC [7].

PD-1 is a type I transmembrane protein that belongs to the immunglobulin B7 superfamily receptors. PD-1 is mainly expressed by T lymphocytes and downstream signaling due to activation by PD-L1 or PD-L2 participates in the coinhibitory regulation of T cell proliferation, activation and proinflammatory cytokine expression [8]. PD-L1, also known as B7-H1 or CD274, is expressed by different immune cell populations including dendritic cells (DC), macrophages as well as T and B cells. However, abberant PD-L1 expression by malignant cells has been identified in several solid tumors and, therefore, constitutes one important immune escape mechanism [9].

The identification of PDAC patient subgroups that benefit from the application of PD-1 or PD-L1 blocking antibodies is not only the key to an effective therapy concept, but also essential for the understanding of tumor biology and treatment adaption in the course of tumor progression. PD-L1 expression status in the tumor might serve as an indicator for therapy response and stratification of patients. However, PD-L1 status is actually a controversially discussed topic since detection and classification of PD-L1 status is not standardized and based on different diagnostic antibodies and threshold definitions [10]. Moreover, it is questionable if the common classification system that only rates PD-L1 expression in tumor but not stromal cells is sufficient for precise prediction and/or prognostic purposes in PDAC therapy. Thus, the aim of this study was to comprehensively characterize PD-L1 expression in stromal and tumoral compartment *via* IHC in a well characterized collective of 59 PDAC tissues and 18 peritumoral pancreatic tissues. For this purpose, we developed a scoring system (POLE Score) that considers PD-L1 expression, in both tumor and stromal cells, in terms of (i) overall PD-L1 expression (P) (ii) cellular origin of PD-L1 (O) (iii) PD-L1 expression in tumor-associated lymph follicles (L) and (iv) enumerated local PD-L1 distribution (E). Finally, we applied this system to the tissue sections and correlated the results with clinic-pathological data as well as findings from IHC studies on markers for proliferation, lymphocyte infiltration and epithelial to mesenchymal transition (EMT) status.

## RESULTS

### Heterogenous PD-L1 expression in tumor tissue from PDAC patients

**Figure 1:**
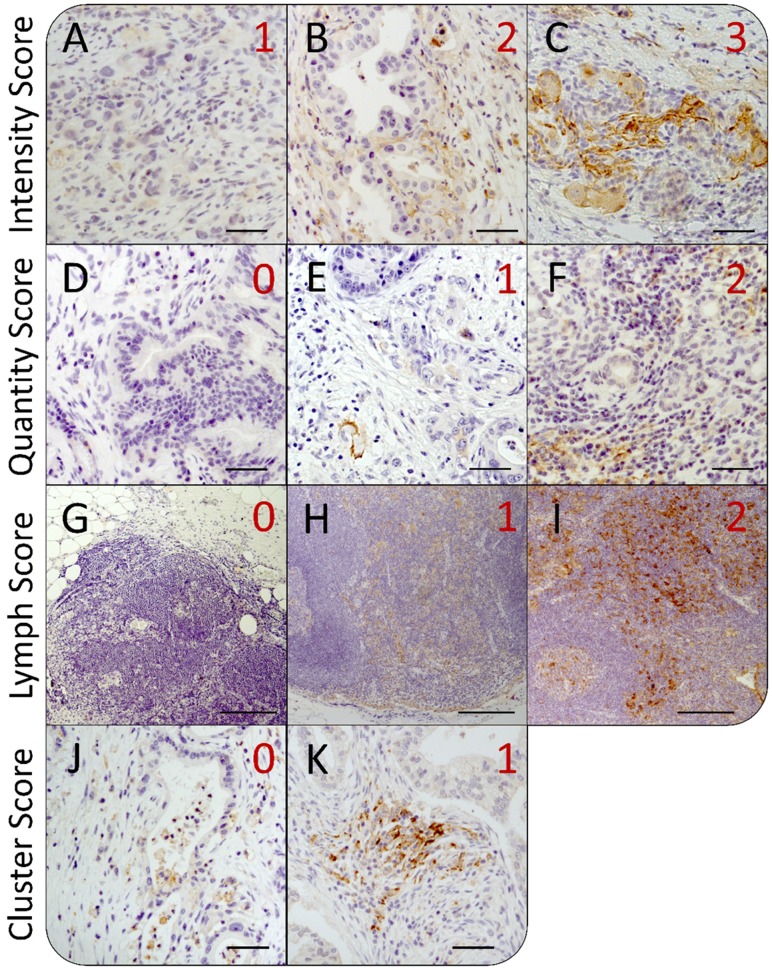
Heterogeneity of intratumoral PD-L1 expression in pancreatic tissue sections from PDAC patients Representative images of immunohistochemical PD-L1 staining in pancreatic tissues of PDAC patients for different scoring values with regard to (**A**–**C**) the staining intensity, (**D**–**F**) the proportion of PD-L1+ cells, (**G**–**I**) the expression in tumor-associated lymph follicles as well as (**J**–**K**) the local distribution of PD-L1+ cells within the tumor. According to the evaluation system, PD-L1 mean staining intensity in fields of view (FoV) showing PD-L1+ cells was rated as (A) weak (1), (B) moderate (2) or (C) strong (3). The proportion of PD-L1+ cells within FoV was scored as (D) negative (0), (E) < 1% PD-L1+ cells (1) or (F) > 1% PD-L1+ cells (2). PD-L1 expression in lymph follicles was rated as (G) negative (0), (H) weak/moderate (1) or (I) strong (2). Finally, distribution of PD-L1+ cells within FoV was categorized as (J) „diffuse/patternless“ (0) and (K) „cluster formation“ (1). Original magnification/scale bar: 100-fold/200 μm (G–I); 200-fold/50 μm (A–F; J–K).

Immunostaining on PD-L1 was performed on whole tumor sections of 59 PDAC patients focusing on PD-L1 expression in neoplastic cells, stromal cells within the desmoplastic reaction as well as tumor-associated lymph follicles (Supplementary Table 1). We identified prominent intra- and intertumoral differences in PD-L1 expression with regard to staining intensity and proportion of PD-L1^+^ cells. Therefore, staining intensities were scored from 1 to 3 (weak, moderate and strong) (Figure 1A–1C) and proportion of PD-L1^+^ cells was rated from 0 to 2 (0%, ≤ 1% and > 1% PD-L1^+^ cells) (Figure 1D–1F) in each microscopic field of view (FoV). Moreover, comparison of PD-L1 expression within tumor-associated lymph follicles with remaining tumor tissue exhibited frequently marked differences. Hence, PD-L1 expression of each tumor-associated lymph follicle was scored separately according to its respective intensity from 0 to 2 (negative, weak, strong) (Figure 1G–1I). Lymph Score was calculated based on the median value of all lymph follicles within the tissue section. Excluding tumor-associated lymph follicles, we observed areas in the tumoral and stromal compartment of PDAC tissues that showed scattered distribution of PD-L1^+^ cells as well as those that exhibited dense clusters of PD-L1^+^ neoplastic and/or stromal cells (Figure 1J, 1K). Thus, the respective pattern within each PD-L1^+^ FoV was rated as 0 (scattered) or 1 (clustered) and Cluster Scores were calculated by mean values of rated FoV within the respective tissue section. Lymph and Cluster scores of PDAC tissue sections ranged from 0 to 2 with a median of 1 (Lymph Score) and from 0 to 0.52 with a median of 0.14 (Cluster Score), respectively.

Although some of the tissues exhibited areas with pronounced PD-L1 expression, the low overall presence of PD-L1^+^ cells in 44 of 56 cases (78.6%) led to Quantity and Intensity Scores of 0. In order to better discriminate between sections with small PD-L1^+^ areas and particularly high PD-L1 expression, an immunreactivity scoring (IRS) system was applied. Therefore, Quantity and Intensity Scores were sumed up for each rated FoV and mean values were calculated for the whole tissue section (Tissue Score). Tissue Scores revealed values in the range from 0 to 2.65 with a mean score of 0.55.

As an additional indicator for overall distribution of PD-L1^+^ cells within the tumor, proportion of FoV graded with an IRS > 0 (%FoV^+^) has been documentated for each tissue section. Proportion of PD-L1^+^ FoV within PDAC tissue sections ranged from 0 to 92.4% with a median proportion of 22.9%. Results from IHC staining evaluation are summarized in Table 1.

**Table 1:** Summary of immunohistochemical staining evaluation of PD-L1 expression in tumor and stromal cells within PDAC tumor and peritumoral tissue sections

	Quantity Score	*n* (%)	Intensity Score	*n* (%)	Tissue Score	*n* (%)	%FoV+	*n* (%)	Lymph Score	*n* (%)	Cluster Score	*n* (%)
Tumor	0 (0%)	44 (78.6)	0 (negative)	44 (78.6)	0	1 (1.8)	≤ 5%	6 (10.7)	0 (negative)	7 (17.9)	0	16 (28.6)
	1 (≤ 1%)	12 (21.4)	1 (weak)	12 (21.4)	< 0.1	4 (7.1)	5% – 10%	6 (10.7)	1 (weak)	17 (43.6)	< 0.1	8 (14.3)
	2 (> 1%)	0 (0)	2 (moderate)	0 (0)	0.1 – < 0.2	4 (7.1)	10% - 20%	13 (23.2)	2 (strong)	15 (38.5)	0.1 – < 0.2	13 (23.2)
			3 (strong)	0 (0)	0.2 - < 0.4	9 (16.1)	20% - < 50%	19 (33.9)			0.2 - < 0.3	9 (16.1)
					0.4 - < 0.8	21 (37.5)	≥ 50%	12 (21.4)			0.3 - < 0.4	7 (12.5)
					≥ 0.8	17 (30.4)					≥ 0.4	3 (5.3)
	Total	56 (100)		56 (100)		56 (100)		56 (100)		39 (100)		56 (100)
	Total/Missing	59 / 3		59 / 3		59 / 3		59 / 3		59 / 20		59 / 3
	**Quantity Score**	***n* (%)**	**Intensity Score**	***n* (%)**	**Tissue Score**	***n* (%)**	**%FoV+**	***n* (%)**	**Lymph Score**	***n* (%)**	**Cluster Score**	***n* (%)**
Peritumoral	0 (0%)	15 (83.3)	0 (negative)	15 (83.3)	0	2 (11.1)	≤ 5%	6 (33.3)	0 (negative)	0 (0)	0	6 (33.3)
	1 (≤ 1%)	2 (11.1)	1 (weak)	3 (16.7)	< 0.1	4 (22.2)	5% – 10%	1 (5.6)	1 (weak)	5 (55.6)	< 0.1	2 (11.1)
	2 (> 1%)	1 (5.6)	2 (moderate)	0 (0)	0.1 – < 0.2	0 (0)	10% - 20%	4 (22.2)	2 (strong)	4 (44.4)	0.1 – < 0.2	4 (22.2)
			3 (strong)	0 (0)	0.2 - < 0.4	4 (22.2)	20% - < 50%	4 (22.2)			0.2 - < 0.3	4 (22.2)
					0.4 - < 0.8	4 (22.2)	≥ 50%	3 (16.7)			0.3 - < 0.4	2 (11.1)
					≥ 0.8	4 (22.2)					≥ 0.4	0
	Total	18 (100)		18 (100)		18 (100)		18 (100)		9 (100)		18 (100)
	Total/Missing	18 / 0		18 / 0		18 / 0		18 / 0		18 / 9		18 / 0

PD-L1 expression is characterized with respect to the proportion of PD-L1^+^ cells (Quantity Score), staining intensity (Intensity Score), local distribution of PD-L1^+^ cells (Cluster Score) as well as expression in lymph follicles (Lymph Score). Immunoreactivity/Tissue Score combines results from Quantity and Intensity Score. PDAC tissue sections were categorized by the proportion of fields of view (FoV) comprising at least one PD-L1^+^ cell (%FoV^+^). Quantity, Intensity as well as Lymph Scores are presented as median and Tissue, %FoV^+^ as well as Cluster Scores as mean values of rated FoV within each tissue. Tissues comprising no neoplastic cells within the respective section but lymph follicles were excluded from Quantity, Intensity, Tissue as well as Cluster scoring and, therefore, denoted as „Missing“.

### PD-L1 expression is higher in tumor tissue of PDAC patients compared to peritumoral pancreatic tissue

**Figure 2:**
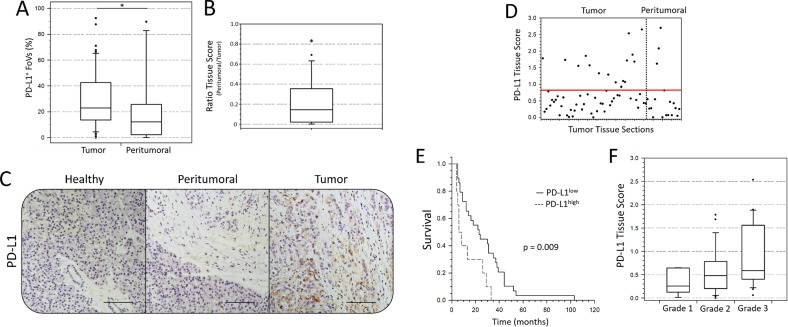
PD-L1 expression is enhanced in PDAC tumor area compared to peritumoral tissue and correlates with clinic-pathological patient characteristics (**A**) Proportion of analyzed fields of view (FoV) with at least one PD-L1+ cell in PDAC tumor and peritumoral tissue sections presented as Box-and-whisker plot. (**B**) Ratio of PD-L1 Tissue Scores in PDAC tumor tissue and respective peritumoral pancreatic tissue presented as Box-and-whisker plot. (**C**) Representative images of immunohistochemical PD-L1 staining in different areas of pancreatic tissue from a PDAC patient. Shown are adjacent healthy acinus tissue (left, “Healthy”), peritumoral margin with desmoplastic stroma (middle, “Peritumoral”) and intratumoral tissue (right, “Tumor”). (**D**) Tissue Scores of each PDAC tumor and peritumoral tissue section are presented as dots by a scatter plot. Red line at a value of 0.8 indicates the identified threshold between PD-L1low and PD-L1high tissue sections. (**E**) Survival LogRank analysis correlates overall survival time (months) of PDAC patients with intratumoral PD-L1 expression (Tissue Score: PD-L1low vs. PD-L1high). Considering surgery-related mortality, patients with survival times of less than 4 months were excluded from analysis. (**F**) PD-L1 Tissue Scores are presented as Box-and-whisker plots with regard to the pathological defined tumor grade. *n* = 56 (D, F); 39 (E). Original magnification/scale bar: 200-fold/100 μm. *n* = 56 vs. 18 (A); 13 (B). ^*^=*p* < 0.05.

In order to examine whether PD-L1 is higher or even exclusively expressed in PDAC tumor tissue compared to peritumoral pancreatic tissue, we analyzed PD-L1 expression within 18 peritumoral tissue sections from PDAC patients. Application of the established scoring system (Table 1) revealed a significantly lower proportion of PD-L1^+^ FoV in peritumoral pancreatic tissue sections compared to tumor tissue sections (12.2% *vs.* 22.9%) (Figure 2A). Accordingly, correlation of PD-L1 expression in the 13 peritumoral tissue sections and their corresponding tumor sections revealed significantly higher Tissue Scores in tumor tissues compared to peritumoral tissues (Tissue Score ratio: 0.144) (Figure 2B, 2C). Notably, 4 of 18 peritumoral tissue sections exhibited comparatively high Tissue Scores (≥ 0.8) and high proportions of PD-L1^+^ FoV (> 30%) (Figure 2D). In comparison to the other peritumoral tissues, these tissue sections exhibited a higher proportion of malignant cells within the tumor margin, indicating that enhanced intratumoral PD-L1 expression in PDAC might be related to the local proximity and proportion of neoplastic cells. Interestingly, in contrast to tumor tissue sections, peritumoral tissues revealed no PD-L1^negative^ lymph follicles. Furthermore, Cluster Scores > 0.4 were exlusively found in neoplastic pancreatic tissues (Table 1).

### PD-L1 Tissue Score correlates with survival and tumor grade in PDAC

Despite the pathological homogeneity within our cohort (98% T3 and 87% T3N1M0), survival markedly differed between patients (0-108 months). Therefore, we examined whether PD-L1 Tissue Score correlates with patient survival. Indeed, we found a significant correlation between a Tissue Score > 0.8 and decreased overall survival (Median survival 23 *vs.* 7 months) (Figure 2E). Moreover, comparison of pathological tumor grade and PD-L1 Tissue Scores revealed that high Tissue Scores tended to be associated with poor tumor differentiation (Tissue Scores (Tumor grade): 0.255 (1) *vs.* 0.481 (2) *vs.* 0.588 (3)) (Figure 2F).

### PD-L1 is predominantly expressed by stromal cells in PDAC

**Figure 3:**
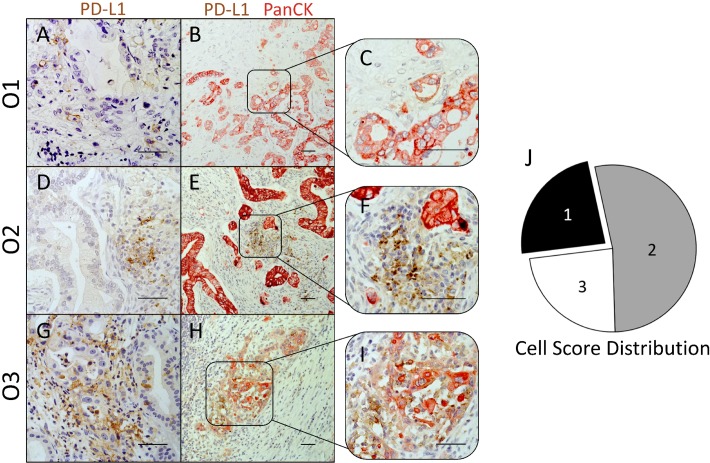
PD-L1 expression in PDAC is not restricted to epithelial/tumor cells, but is localized to a large extent within the tumor stroma (**A**–**I**) Representative images of immunohistochemical PD-L1 (brown)/PanCK (red) stainings in PD-L1high graded pancreatic tissues of PDAC patients. Cellular origin of PD-L1 expression observed in single stainings (A, D and G) was evaluated in PD-L1/PanCK double stainings (B and C, E and F, H and I). Tissues were classified into 3 groups with regard to the prevalent cellular origin of PD-L1 expression: Cell Score 1 (PanCK+ PD-L1+), Cell Score 2 (PanCK- PD-L1+) and Cell Score 3 (PanCK± PD-L1+). (**J**) Pie chart illustrates the proportion of PDAC tissues rated as Cell Score 1 (black), 2 (grey) and 3 (white) within PD-L1high graded tissues. Original magnification/scale bar: 200-fold/100 μm (B, E and H); 400-fold/100 μm (A, C, D, F, G and I).

In the concept of PD-1/PD-L1 targeting therapy, aberrant PD-L1 expression by malignant cells is regarded as a crucial immune escape mechanism [11]. However, also stromal PD-L1 expression might contribute to the immunosuppressive microenvironment in PDAC. In order to discriminate tumoral- and stromal-associated PD-L1 expression, we performed PD-L1/PanCK IHC staining of PD-L1^high^ classified PDAC tissue sections. Cell Scores resulting from this analysis indicate whether PD-L1 is primarily expressed by epithelial/cancer cells (Cell Score 1; Figure 3A–3C), stromal cells (Cell Score 2; Figure 3D–3F) or whether PD-L1 is expressed by both populations to a similar extent (Cell Score 3; Figure 3G–3I). Interestingly, examined Cell Scores revealed that PD-L1 is not predominantly expressed by cancer cells but rather by stromal cells. In detail, only 4 cases (23.5%) showed PD-L1 expression exclusively in PanCK^+^ cells (Cell Score 1). In contrast, 53% of PD-L1^high^ classified tissue sections exhibited PD-L1 expression primarily in stromal cells (Cell Score 2). In 5 of these tissue sections PD-L1 was also expressed by a small proportion of PanCK^+^ cells (< 10% of all PD-L1^+^ cells). Additionally, in 4 PDAC tissues (23.5%) PD-L1 was highly expressed by both PanCK^+^ and PanCK^−^ cells (Cell Score 3, Figure 3J).

### Improved characterization of PD-L1 expression status in PDAC by POLE Score

In order to consider all information obtained from PD-L1 expression analyses, we developed a comprehensive characterization score that resembles the TNM staging system. For this purpose, compiled Tissue, Cell, Lymph and Cluster Scores were converted into a four-letter code (POLE Score) that characterizes intratumoral overall PD-L1 expression (P), cellular Origin of expressed PD-L1 (O), PD-L1 expression status in tumor-associated Lymph follicles (L) and the Enumerated local distribution of PD-L1^+^ cells (E) by single digits. Characterization criteria of each POLE Score parameter are summarized in Table 2.

**Table 2:** POLE Score criteria

Values and criteria					
Parameter	Description	0	1	2	X
P	PD-L1 intratumoral overall expression	Tissue Score = 0	Tissue Score < 0.8	Tissue Score > 0.8	no ratable
		or %FoV PD-L1+ ≤ 20%	and %FoV PD-L1+ > 20%		structures
	Cellular Origin of PD-L1	1	2	3	X
O		Cell Score = 1	Cell Score =2	Cell Score =3	not rated
					(P0 or P1)
	PD-L1 Expression in intratumoral Lymph follicles	0	1	2	X
L		Lymph Score = 0	0 < Lymph Score < 2	Lymph Score = 2	no ratable
					structures
	Enumerated PD-L1 expression (local scattering)	0	1	X	
E		Cluster Score < 0.4	Cluster Score ≥ 0.4	no ratable	
				structures	

**Figure 4:**
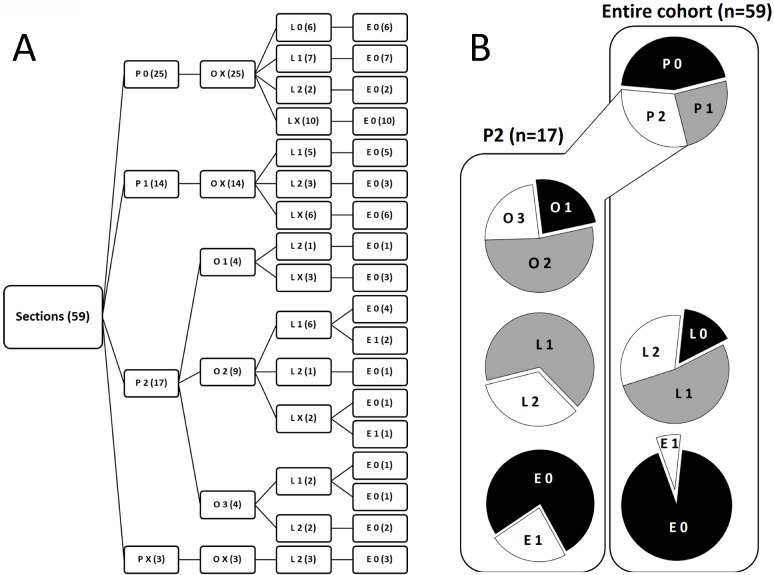
Characterization of PD-L1 expression in PDAC by POLE Score system (**A**) Tree diagram illustrates the distribution of POLE Scores for PD-L1 expression in pancreatic tissue sections of PDAC patients. Scoring values P0-2 (overall PD-L1 expression), O1-3 (PD-L1 cellular Origin), L0-2 (PD-L1 expression in Lymph follicles) and E0-1 (Enumerated distribution of PD-L1+ cells) are depicted at the end of the branches. Number of cases rated for the score within the respective branch are indicated in brackets. (**B**) Pie charts illustrate the proportion of compiled POLE scores within the entire cohort (right, *n* = 59) and subset of P2 scored (left, *n* = 17) PDAC tissues. X = not rated (excluded in pie charts).

According to these criteria, collected data on PD-L1 expression within our cohort of PDAC tissues were converted into respective POLE Scores (Figure 4A). P-Score proportion indicated that PD-L1 is markedly expressed in 30.4% (P2) of PDAC tissues, while it is low expressed in 25.0% (P1) and absent or almost absent in 44.6% (P0) of the specimens (Figure 4B). Notably, 76.5% of P2 rated tissues showed either almost exclusive (O2, 53.0%) or prominent (O3, 23.5%) PD-L1 expression within the stroma (Figure 4B). In comparison to the entire cohort, P2 graded tissues revealed no PD-L1^−^ lymph follicles (Figure 4B). Moreover, only P0 scored tissues comprised sections rated L0 (Figure 4A). Finally, all PDAC tissues markedly enriched for PD-L1^+^ cell clusters (E1) belonged to the P2 classified subgroup (Figure 4B).

### Correlation of PD-L1 expression and tumor stromal composition

Since the present cohort of PDAC tissues has been already extensively characterized regarding tumor stromal composition, proliferation and EMT status, we correlated available data from previous immunohistochemical studies [12] with POLE Scores (Supplementary Table 2). For this purpose, POLE Scores (except for E-Score) were dichotomized prior to statistical analyses into:

I) +P 1/2 (low overall PD-L1 (P0 + P1) *vs.* high overall PD-L1 (P2))

II) +O 1/2 (only PanCK^+^ cells PD-L1^+^ (O1) *vs.* high stromal PD-L1 (O2 + O3))

III) +L 1/2 (PD-L1^−^ lymph follicles (L0) *vs.* PD-L1^+^ lymph follicles (L1 + L2))

**Figure 5:**
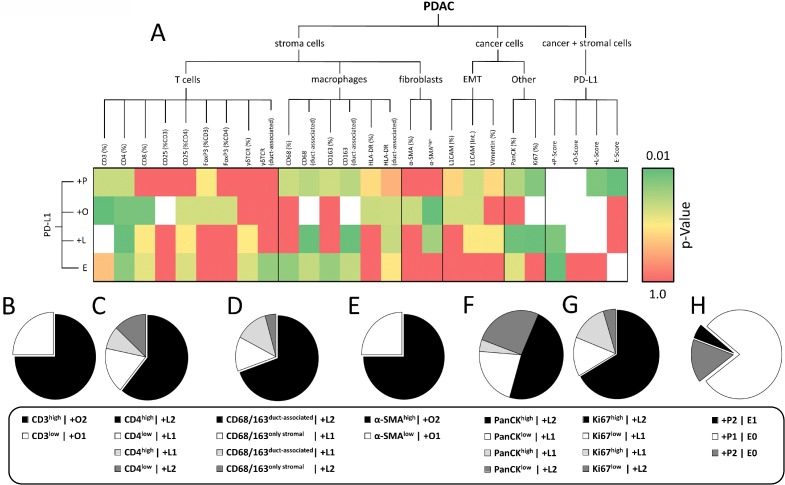
PD-L1 expression characterized by POLE Score correlates with intratumoral proportion of distinct immune cell populations (**A**) Heatmap illustrates results from statistical correlation of dichotomized POLE Scores, markers for stromal cell populations, EMT and Ki67 status assessed by immunohistochemical stainings. Dichotomization was performed according to the following pattern: +P1 (=P0+P1) vs. +P2 (=P2); +O1 (=O1) vs. +O2 (=O2+O3); +L1 (=L0) vs. +L2 (=L1+L2). *P*-values from statistical analyses are illustrated by color gradient (dark green: *p* = 0.01 to dark red: *p* = 1.0; white: statistical analysis not possible). Pie charts show proportion of subgroups within correlations of (**B**) +O-Score vs. CD3 (% stroma), (**C**) +L-Score vs. CD4 (% stroma), (**D**) +L-Score vs. CD68/CD163 (duct-associated), (**E**) +O-Score vs. α-SMA (intensity), (**F**) +L-Score vs. PanCK (% section), (**G**) +L-Score vs. Ki67 (% epithelium) and (**H**) +P-Score vs. E-Score. Statistical analyses were performed by Chi-square/Fisher exact test.

Statistical correlation of dichotomized POLE Scores with markers related to the infiltration of T cells (CD3^+^), cytotoxic T cells (CD8^+^), regulatory T cells (CD25^+^, FoxP3^+^) and γδ T cells (γδ TCR^+^) revealed significant interrelations between +O-Score and T cell infiltration (CD3^+^) as well as +L-Score and presence of CD4^+^ T cells (Figure 5A). In detail, all PDAC tissues showing high stromal PD-L1 expression were highly infiltrated by CD3^+^ T cells. Conversely, all specimens with PD-L1 expression restricted to PanCK^+^ cells were characterized by a low abundance of CD3^+^ T cells (Figure 5B). Likewise, PD-L1 expression in lymph follicles positively correlated with presence of CD4^+^ T cells. Thus, 18 of 23 cases (78.2%) showed either +L1/CD4^low^ or +L2/CD4^high^ status (Figure 5C). Moreover, we identified significant coherences between PD-L1 expression in tumor-associated lymph follicles and the local abundance of tumor-associated macrophages (TAMs) (CD68^+^, CD163^+^) (Figure 5A, 5D). In detail, 16 of 23 cases (69.5%) showed +L2 status associated with high proportion of CD68^+^ or CD163^+^ cells in close proximity to epithelial cells (duct-associated^+^) (Figure 5D). Indicating a link between cellular origin of PD-L1 expression and presence of myofibroblasts, we found an exlusive correlation between +O1/α-SMA^low^ and +O2/α-SMA^high^ rated specimens, respectively (Figure 5E). Interestingly, the proportion of PanCK^+^ cells and Ki67^+^ epithelial cells in PDAC tissues tended to correlate with respective +L-Scores (*p* = 0.069; *p* = 0.053) (Figure 5A). In detail, 16 of 23 cases revealed either +L1/PanCK^low^ or +L2/PanCK^high^ status and 17 of 23 cases exhibited either +L1/Ki67^low^ or +L2/Ki67^high^ status (Figure 5F, 5G). Closer examinations on serial tissue sections demonstrated that absent/low PD-L1 expression in stroma or lymph follicles was linked to Ki67^−/low^ status of neoplastic ducts while areas with marked stromal or lymph follicle-associated PD-L1 expression showed high proportion of Ki67^+^ epithelial/cancer cells (Supplementary Figure 1). Finally, PDAC tissue sections exhibiting prominent formation of PD-L1^+^ cell clusters (E1) were only found within the +P2 subgroup. Accordingly, 30 of 36 E0 rated specimens showed low overall PD-L1 expression (Figure 5H).

Taken together, our data indicate that PD-L1 expression in PDAC markedly varies in terms of different tumor compartments (neoplastic vs. stromal cells vs. tumor-associated lymph follicles) and correlates with distinct patterns of tumor/stromal composition.

### PD-L1 expression in PDAC is markedly increased within particular tumor regions and associated with distinct stromal composition

**Figure 6:**
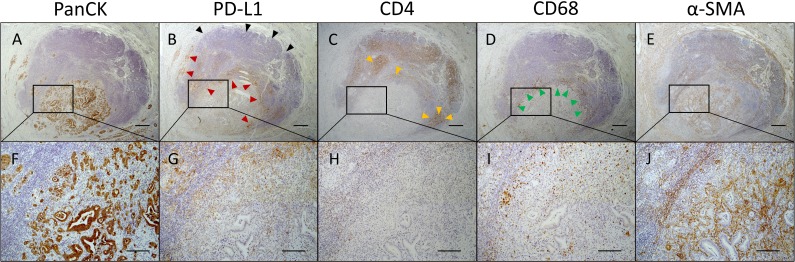
Polarized PD-L1 expression at tumor-lymph follicle interface coincides with the presence of CD4+ T cells and CD68+ macrophages Representative images of (**A**, **F**) pan-cytokeratin (PanCK), (**B**, **G**) PD-L1, (**C**, **H**) CD4, (**D**, **I**) CD68 and (**E**, **J**) α-SMA immunohistochemical stainings in serial pancreatic tissue sections from a PDAC patient. (B) Shown is an infiltrated tumor-associated lymph follicle exhibiting high proportion of PD-L1+ cells at the tumor margin (red arrow heads) and absence of PD-L1 expression at the tumor-averted border (black arrow heads). PD-L1 expression is co-localized with (C) CD4 (yellow arrow heads) and (D) CD68 expression (green arrow heads). Original magnification/scale bar: 25-fold/500 μm (overviews; A–E); 100-fold/200 μm (detail; F–J).

Based on the results from statistical analyses, we compared PD-L1 enriched areas with CD3^+^, CD4^+^, CD8^+^, CD68^+^, CD163^+^, α-SMA^+^ and PanCK^+^ structures in serial tissue sections identifying two particular areas frequently enriched for PD-L1^+^ cells in PDAC tissues. The first type of area showed high PD-L1 expression at the tumor-lymph follicle interface as depicted in Figure 6B, 6G. Here, the tumor mass surrounded by a lymph follicle showed high proportion of PanCK^+^ neoplastic cells (Figure 6A, 6F) as well as high α-SMA expression (Figure 6E, 6J) and peripheral accumulation of CD68^+^ cells (Figure 6D, 6I). Interestingly, PD-L1 expression appeared to be polarized towards adjacent neoplastic cells. Thus, tumor-lymph follicle interface was highly enriched for PD-L1^+^ cells but not tumor-averted border (Figure 6B, arrow heads). These findings are in line with data from statistical analyses revealing a correlation between PanCK expression and L-Score by trend. Further supporting results from statistical analyses, CD4^+^ clusters inside the lymph follicle showed overlapping areas with PD-L1 staining while being almost absent within the tumor (Figure 6C, 6H, arrow heads). Notably, PD-L1 expression at the tumor-lymph follicle interface seemed to largely coincide with the presence of CD68^+^ cells (Figure 6B, 6G ***vs.*** 6D, 6I, arrow heads) indicating that TAMs highly express PD-L1.

**Figure 7:**
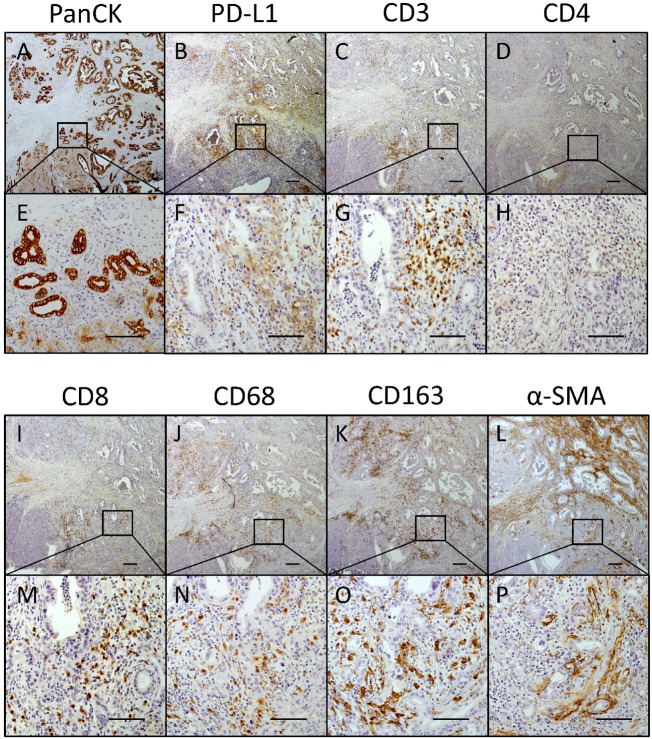
PD-L1 is markedly expressed within tumor areas that show high proportion of CD8+ T cells, macrophages and myofibroblasts Representative images of (**A**, **E**) PanCK, (**B**, **F**) PD-L1, (**C**, **G**) CD3, (**D**, **H**) CD4, (**I**, **M**) CD8, (**J**, **N**) CD68, (**K**, **O**) CD163 and (**L**, **P**) α-SMA immunohistochemical stainings in serial pancreatic tissue sections of a PDAC patient. Original magnification/scale bar: 25-fold/200 μm (overviews; A–D and I–L); 200-fold/100 μm (detail; E–H and M–P).

The second type of area that frequently showed a high amount of PD-L1^+^ cells was located within tumor regions comprising neoplastic cells in close proximity to remaining acinus tissue (Figure 7A, 7E) associated with high abundance of CD3^+^ and CD68^+^ immune cells and low proportion of myofibroblasts (α-SMA). Figure 7 shows several areas with high PD-L1 expression (Figure 7B, 7F) at the tumor margin. Notably, PD-L1 is expressed within these areas by both tumor and stromal cells. Underlining our results from statistical analyses, these areas exhibited high proportions of CD3^+^ T cells (Figure 7C, 7G) being not CD4^+^ (Figure 7D, 7H) but CD8^+^ T cells (Figure 7I, 7M). Furthermore, CD68^+^ macrophages and especially CD163^+^ subpopulations were found to be tightly associated with pancreatic cancer cells (Figure 7J, 7N, 7K, 7O). Interestingly, α-SMA expression within these areas was low in comparison to overall intratumoral α-SMA expression (Figure 7L, 7P; compare upper area *vs.* marked area).

In summary, our analyses on serial tissue sections highlighted that PD-L1 expression in PDAC is very heterogenous, restricted to locally defined intratumoral areas and associated with a high infiltration of CD8^+^ effector T cells and CD68^+^CD163^+^ macrophages. Hence, these findings substantiate our results from statistal analyses.

### Tumor-associated macrophages represent a PD-L1 expressing stromal cell population in PDAC

**Figure 8:**
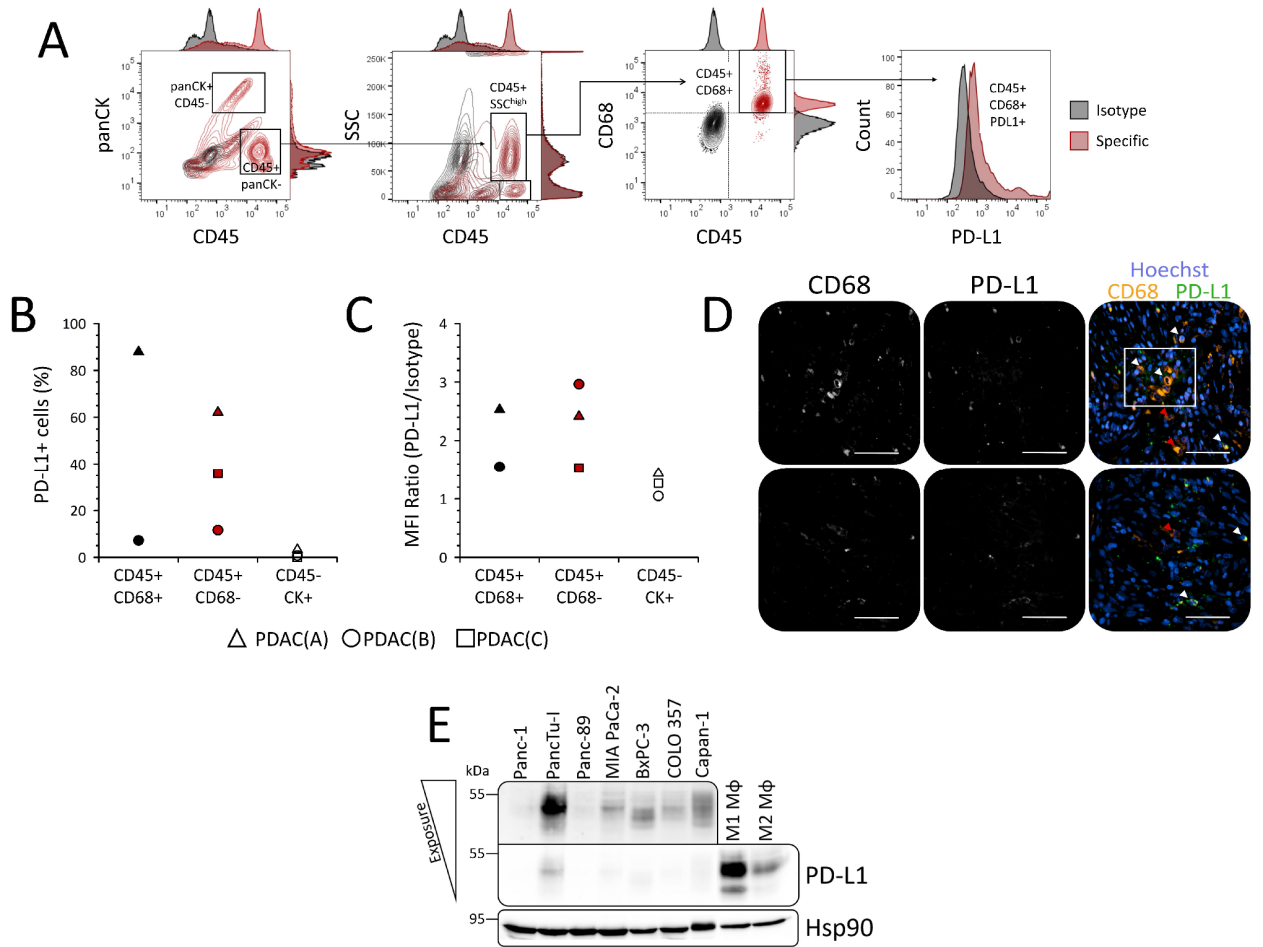
Tumor-associated macrophages represent a PD-L1 expressing stromal cell population in PDAC patients and distinct tumor areas (**A**) Gating strategy for detection of cell surface PD-L1 expression in distinct populations of freshly isolated cells from resected PDAC specimens by flow cytometry. Epithelial/tumor cells were discriminated from leukocytes by panCK/CD45 staining. Tumor-associated macrophages (TAMs) within the CD45+ leukocyte populations were discriminated from e.g. lymphocytes via side scatter (SSC) and CD68 expression. Specificity of stainings (red contour plots/histograms) was ensured by parallel detection of signals from respective isotype stainings (black contour plots/histograms). (**B**, **C**) Scatter plots summarize results from flow cytometric detection of PD-L1 cell surface expression in freshly isolated TAMs (CD45+ CD68+; black symbols), non-TAM leukocytes (CD45+ CD68-; red symbols) and epithelial/tumor cells (CD45- panCK+; white symbols) of PDAC specimens from three different patients with regard to (B) proportion of PD-L1+ cells and (C) median fluorescence intensity (MFI) ratio (MFI PD-L1/MFI Isotype). (**D**) Representative images of CD68 and PD-L1 immunofluorescence co-stainings in PDAC tissue sections from two different patients. Shown are grey scale signals from single channels for detection of CD68 (left) and PD-L1 (middle) staining as well as overlay (right) of CD68 (orange), PD-L1 (green) and nuclei staining by Hoechst (blue) for analysis of co-localization. Marked are PD-L1+ CD68+ (white arrow heads) and PD-L1- CD68+ (red arrow heads) TAMs as well as a cluster of PD-L1+ CD68+ TAMs (white quadrant). Original magnification/scale bar: 200-fold/50 μm. (**E**) Representative western blot of whole-cell lysates from various PDAC cell lines (Panc-1, PancTu-I, Panc-89, MIAPaCa-2, BxPC-3, COLO357) and *in vitro* differentiated human macrophages. Macrophages were generated by stimulation of isolated monocytes from healthy donors with either 50 ng/mL GM-CSF (M1 Mφ) or M-CSF (M2 Mφ) for 7 days. Hsp90 was detected as loading control. Upper box shows PD-L1 signal from PDAC cell line lysates after increased light exposure.

Since immunohistochemical stainings in serial PDAC tissue sections revealed a high abundance of CD68^+^ and CD163^+^ TAMs within tumor areas with marked PD-L1 expression, our data suggest that TAMs represent one of the PD-L1 expressing stromal populations. For confirmation, multicolour flow cytometric analysis was performed with freshly isolated cells from enzymatic dissociated PDAC specimens. Our established staining panel consisting of antibodies against panCK, CD45, CD68 and PD-L1 allowed the identification of TAMs via gating on panCK^−^ CD45^+^ SSC^high^ CD45^+^ cells as well as the detection of cell surface expressed PD-L1 within this population (Figure 8A). Flow cytometric analyses of tumor specimens from three PDAC patients well reflected the described tumor heterogeneity of PD-L1 expression in PDAC. TAMs could be detected in two specimens: one of these specimens exhibited a TAM population mainly composed of PD-L1^+^ cells (87.9 %) (Figure 8B) with a median fluorescence intensity ratio of 2.53 (Figure 8C) indicating high cell surface expression levels. In contrast, the other specimen revealed a minor population of PD-L1^+^ TAMs (7.2 %) (Figure 8B) and lower median fluorescence intensity ratio (1.55) (Figure 8C). Furthermore, PD-L1 expression by CD68^+^ TAMs was validated by double immunofluorescence stainings of PDAC tissue sections clearly showing that CD68+ cells express PD-L1 (Figure 8D). However, similar to the flow cytometric analysis immunofluorescence stainings also revealed that not all TAMs express PD-L1 indicating that other stromal cells also account for PD-L1 expression in PDAC.

In line with our previous results, these findings also demonstrate that only a small proportion of panCK^+^ cells showed, if any, comparatively low cell surface PD-L1 expression (Figure 8B: 3.27 %, 0.78 % and 0.0%). Further supporting these data, western blot analyses showed that PD-L1 expression is nearly absent in PDAC cells but pronounced in *in vitro* differentiated macrophages (Figure 8E). These findings also revealed that both GM-CSF and M-CSF are potent inducers of PD-L1 expression in monocytes and macrophages (Figure 8E). Notably, GM-CSF differentiated macrophages being characterized by a rather pro-inflammatory phenotype (M1 Mφ) exhibited considerably higher PD-L1 expression levels than M-CSF differentiated anti-inflammatory macrophages (M2 Mφ).

Altogether, these findings support the view that PDAC cells rarely express high PD-L1 levels at the cell surface while TAMs represent one stroma cell population acccounting for high PD-L1 expression in PDAC.

## DISCUSSION

Restoring anti-tumoral immune response by interference with coinhibitory PD-1/PD-L1 signaling currently represents one of the most promising and most intensively investigated strategies in cancer therapy [13–15]. However, although the theory behind its mode of action seems to be simple, the tumor-specific characteristics that either favor or impede effective PD-1/PD-L1 targeting are insufficiently understood. In this context, a major challenge is to reliably identify patients that benefit from PD-1/PD-L1 blockade. While several preclinical studies proposed PD-L1 expression as a predicative marker, correlations between treatment responses and respective tissue based PD-L1 status in clinical trials are still poor, absent or contrary [16–19]. Moreover, assessment of PD-L1 status itself is controversially discussed, since neither detection systems nor classification criteria are standardized [10]. Underlining the questionable significance of current methods for PD-L1 status assessment, results from phase I of the ongoing Blueprint study indicate that available assays that only rate PD-L1 expression by neoplastic cells lead to contrary classifications of PD-L1 status in 37% of the cases [20]. As a result, current indications for application of PD-1/PD-L1 blocking antibodies are either non-existent or failure of standard oncological treatment regimens in most cases.

To our knowledge, there are at least nine others studies that reported PD-L1 expression in PDAC [21–29]. However, this study is the first which provides a comprehensive charaterization (POLE Score) that considers PD-L1 expression by tumor and stromal cells in PDAC. We identified three subgroups within our cohort that were categorized into absent (P0), low (P1) and high (P2) PD-L1 expression. Supporting this finding, Birnbaum *et al.* identified a similar proportion of cases exhibiting high PD-L1 expression on mRNA level within a cohort of 453 pancreatic cancer tissues [26]. In general, recent data suggest that prominent PD-L1 expression in PDAC is restricted to a minor subgroup of tumors. Notably, PD-L1 expression seems not to correlate with pathological staging, since we detected marked differences within our homogenous cohort (87% T3N1M0). However, in line with the results by Wang *et al.* and Geng *et al.*, we showed that PD-L1 expression rather correlates with tumor grade, indicating a link between loss of tumor differentiation and immune regulation [22, 25]. With regard to the prognostic significance of our POLE Score, we and others found a reduced overall survival in the subgroup of patients with PD-L1^high^ classified tumors compared to those with PD-L1^low^ tumors [21, 26]. In contrast, other studies reported a better prognosis in terms of overall or progression-free survival associated with high PD-L1 expression in PDAC and other malignancies [27, 28, 30]. Still, survival correlations in PDAC need to be examined critically, since recovery from surgery and the presence of clinically non-detectable micrometastases in tumors classified as M0 or resected as R0 represent unknown variables that massively affect clinical outcome [31–33].

One of the key innovations of our study is the discrimination between PD-L1^+^ neoplastic and stromal cells. Demonstrating that PD-L1 is predominantly expressed by stromal cells, we underline that common classifications considering only PD-L1^+^ tumor cells exclude a major population of PD-L1^+^ cells in PDAC that might have tumor biological and therapeutical significance. In line with these observations and supporting the clinical importance of stromal PD-L1 expression, several pre-clinical studies highlighted the presence and prognostic relevance of PD-L1 expressing immune cell populations [34–40]. Here, we show that accumulation of PD-L1^+^ tumor and stromal cells is associated with local high infiltration of CD8^+^ T cells and CD68^+^/CD163^+^ TAMs. These findings are in line with previous reports by Thompson and colleagues as well as Knudsen *et al.* who showed similar correlations between PD-L1 expression and local infiltration of CD163^+^ macrophages and/or CD8^+^ T cells in human gastric adenocarcinoma and PDAC, respectively [35, 36]. Local PD-L1 expression might be induced by CD8^+^ T cell secreted IFN-γ being one of the most potent inducers of PD-L1 [36, 41]. Moreover, our *in vitro* studies showed that GM-CSF and M-CSF differentiated M1- and M2-like polarized macrophages exhibit considerable PD-L1 expression and that GM-CSF secreted by T cells and fibroblasts but also cancer cells enhance PD-L1 expression in monocytes (unpublished data). This might also explain the fact that our data suggest the local proximity of malignant cells within the desmoplastic tumor margin as a requisite for accumulation of PD-L1^+^ stromal cells. Furthermore, since CD163 is a prominent marker of anti-inflammatory or M2-like polarized TAMs, recruited M2-like polarized TAMs might impede potent effector T cell responses. In this context, we and others previously reported on the role of M2-polarized TAMs in tumor progression of PDAC [42–44]. Supporting our hypothesis of local CD8^+^ T cell response suppression by TAM-associated PD-L1 expression, both Zhang *et al.* and Kleinovink *et al.* showed in murine models of PDAC and colon carcionoma that myoleid cells substantially contribute to tumor initiation, growth of established tumors and suppression of CD8^+^ T cell response *via* self and induction of tumor cell-associated PD-L1 expression [45, 46]. Nevertheless, we also identified cases in which PD-L1 expression was exclusively restricted to tumor cells. Supporting the assumption that genomic alterations acquired during malignant transformation might cause the ability for aberrant PD-L1 expression in pancreatic ductal epithelial cells, we showed that ducts in peritumoral tissues lack PD-L1 expression and that most PDAC cell lines were characterized by low PD-L1 expression. However, not all PDAC cell lines responded towards IFN-γ stimulation with enhanced PD-L1 expression (unpublished data). Therefore, results from other studies suggest that malignant cells expressing PD-L1 might play a central role in distinct processes of tumor progression, e.g. dissemination of circulating tumor cells and metastasis [47].

Finally, our findings also suggest that PD-1/PD-L1-mediated signaling in PDAC constitutes an immunosuppressive mechanism that is predominantly restricted to the tumor margin and tumor-lymph follicle interface but not the whole tumor. In line with preliminary results from an ongoing phase 2 clinical trial using PD-L1 antagonist Durvalumab in monotherapy for the treatment of metastatic PDAC (NCT02558894), our results implicate that the clinical benefit of therapeutically targeting PD-1 or PD-L1 in monotherapy might be limited. Therefore, it has to be considered that PD-L1 represents just one of several immunoregulatory molecules within the immunosuppressive stroma of PDAC and further studies are needed to unravel the clinical relevance of tumor- and stromal-associated PD-L1 expression within this complex network.

## CONCLUSIONS

This study provides a detailed characterization of PD-L1 expression in PDAC and indicates that PD-L1 expression and its tumor biological relevance have to be rated context dependent, but individual consideration of PD-L1 status might not be applicable for stratification of PDAC patients. Applying the POLE Score, we identified strong correlations between specifically locally restricted PD-L1 expression and stromal composition. Notably, we showed that PD-L1 is predominantly expressed by stromal cells in PDAC, a factor whose role in both tumor biology and immunotherapy is poorly understood, yet. However, validation of our POLE scoring system in an independent PDAC cohort and future clinical trials have to evaluate whether POLE Score might serve as a predictive marker for efficient immunotherapy in PDAC.

## MATERIALS AND METHODS

### Ethics statement

The research was approved by the ethics committee of the University Hospital Schleswig-Holstein (reference number: D430/09). Written informed consent was obtained from all patients.

### Tissues & study population

Pancreatic tissues were obtained from patients during oncological surgery. Gross sectioning, specimen embedding and histopathological diagnosis were done by board certified surgical pathologists in the Institute of Pathology, UKSH Campus Kiel. The selection of tissue blocks was exclusively based on the presence of tumor cells in the PDAC tissue. Moreover, 18 peritumoral tissue sections of PDAC patients were analyzed of which 13 corresponded to the available tumor tissues. Clinic-pathological data were obtained from the hospital records and the *Epidemiological Cancer Registry* of the state of Schleswig-Holstein, Germany. All patient data were pseudonymized prior to study inclusion. Patient characteristics are summarized in Supplementary Table 1.

### Histology

Tissue specimens were fixed in formalin and embedded in paraffin (FFPE). Deparaffinized sections were stained with hematoxilyn and eosin. Tumor grade and stage were classified according to the 7th edition of the UICC guidelines.

### Immunohistochemistry

Immunohistochemical PD-L1 stainings of pancreatic FFPE tissue sections were carried out with a Bondmax automated slide staining system, using the Polymer Refine Detection Kit (both Leica Biosystems, Wetzlar, Germany) and a rabbit monoclonal anti-PD-L1 antibody (8.76 μg/ml, clone #E1L3N, Cell Signaling, Frankfurt a.M., Germany). Specificity of the staining was ensured by application of a respective rabbit IgG isotype control revealing no staining. All other stainings were performed and are described in a former study [12].

### Evaluation of PD-L1 staining

Pancreatic tissue sections of PDAC patients were immunohistochemically stained with a monoclonal antibody against PD-L1 and screened at 200-fold magnification using an Axioplan 2.0 microscope (Zeiss, Jena, Germany). Since whole tissue sections (~ 1-6 cm^2^ area) were analyzed, evaluation of each tumor comprised up to 800 microscopic fields to properly consider tumor heterogeneity. Each field of view (FoV) (Diameter = 0.87 mm at 200-fold magnification) was rated regarding the percentage of PD-L1 positive cells and the mean staining intensity of stained cells. While for PD-L1 expression in tumor cells only the membranous staining was evaluated, stromal cells were rated positive when stained regardless of the cellular PD-L1 localisation. Furthermore, only ductal epithelial, tumor and stromal cells were included in the rating, while fat tissue, neural and acinus cells were excluded. Lymph follicles were rated seperately. The following immunoreactivity scoring system (IRS) was applied for each FoV: (I) the presence and percentage of PD-L1^+^ cells were graded as 0 (negative), 1 (≤ 1% positive) or 2 (> 1% positive), (II) the mean staining intensity of FoV comprising PD-L1^+^ cells were graded as 1 (weak), 2 (moderate) or 3 (strong). Total number of graded FoV depended on the size of the tumor tissue section (30-600). Hereafter, the IRS for each FoV was calculated by summation of both scores resulting in values ranging from 0 to 5. Finally, the „Tissue Score“ for each specimen was determined by calculating the mean value of its total IRS scores. In order to characterize the scattering of PD-L1 positive cells within each tissue section, the proximity of PD-L1 expressing cells towards each other within FoV with an IRS >0 was graded as 0 (PD-L1 positive cells are widely distributed) or 1 (formation of PD-L1^+^ cell cluster). The resulting „Cluster Score“ for each specimen represents the mean value of all rated FoV resulting in values between 0 and 1. In order to score PD-L1 expression in tumor-associated lymph follicles, staining intensity within each follicle was graded as 0 (negative), 1 (weak) or 2 (strong). The resulting „Lymph Score“ for each specimen represents the median value of all rated lymph follicles. Correlation of Tissue Scores with overall survival resulted in classification of PD-L1^low^ and PD-L1^high^ tissue sections. Hereafter, proportion of PD-L1^+^ PanCK^+^ and PD-L1^+^ PanCK^−^ cells within PD-L1^high^ tissue sections was evaluated by immunohistochemical PanCK/PD-L1 staining. Tissue sections were screened at 400-fold magnification to count the amount of PanCK^+^ PD-L1^+^ and PanCK^−^ PD-L1^+^ cells within each FoV Diameter = 0.46 mm at 400-fold magnification. FoV comprising more PanCK^+^ PD-L1^+^ cells were graded as 1 and FoV comprising more PanCK^−^ PD-L1^+^ cells were rated as 2. Cell Scores 1 (≥ 90% FoV rated as 1), 2 (≥ 90% FoV rated as 2) and 3 (∑FoV=1 ≈ ∑FoV=2) are based on the overall proportion of FoV graded as 1 or 2 within the tissue section. All evaluations were performed twice in a blinded manner. In case of discrepant results, sections were additionally evaluated by a second investigator.

### Isolation of cells from fresh PDAC tissues and flow cytometric analysis

Fresh tumor tissues were obtained from PDAC patients during surgery at the Community Hospital in Kiel. Histopathological diagnosis was performed by board certified surgical pathologists at the Institute of Pathology of the Community Hospital in Kiel. Isolation of vital tumor and stromal cells from PDAC tissues was performed with the Tumor Dissociation Kit (human) (Miltenyi Biotec, Bergisch-Gladbach, Germany). Briefly, tumor tissue was cut with a scalpel into pieces of approximately 0.5 mm^3^ in size and incubated in 10 ml RPMI 1640 supplemented with an enzyme mix provided by the kit for 1 h at 37° C on a roller mixer. Afterwards, remaining undissociated tissue pieces were filtered out with cell strainers (mesh sizes: 100 μm and 30 μm) and the filters were washed with 20 ml RPMI 1640. After centrifugation at 300 × g for 10 min at 4° C, cells were washed in cold MACS buffer (PBS supplemented with 2% FCS and 1 mM EDTA) and counted. Before staining for flow cytrometric analysis, Fc receptor blocking was performed with FcR Blocking Reagent (human) (Miltenyi Biotec) according to the manufacturer's instructions. For extracellular staining of cell surface proteins, 1–5 × 10^5^ cells were incubated for 30 min at 4° C with following fluorochrome-conjugated antibodies: anti-human CD45-BV510 (clone #HI30, 4 μg/ml) (Biolegend, Fell, Germany) and anti-human PD-L1-PE-Cy7 (clone #MIH1, 20 μg/ml) (BD Bioscience, Heidelberg, Germany). Afterwards, cells were washed twice and subjected to permeabilization with Intracellular Fixation and Permeabilization Buffer Set (Thermo Scientific, Schwerte, Germany) for intracellular staining according to the manufacturer's instructions. For intracellular staining, cells were cells were incubated for 20 min at 4° C with following fluorochrome-conjugated antibodies: anti-human cytokeratin-FITC (clone #CK3-6H5, 0.4 μg/ml) (Miltenyi Biotec) and anti-human CD68-APC (clone #Y1/82A, 0.5 μg/ml) (Biolegend). Finally, cells were washed twice and stored in MACS buffer supplemented with 1% PFA until analysis with an LSRFortessa flow cytometer (BD Bioscience). Staining specificities were verified by stainings with respective isotype controls and compensation was adjusted with single stainings. Evaluation was performed with FlowJo v10 (FlowJo LCC, Oregon, US).

### Immunofluorescence staining

Serial FFPE tissue sections from PDAC patients that exhibited high PD-L1 expression in previous IHC stainings were subjected to immunofluorescence co-staining for CD68 and PD-L1. Briefly, FFPE tissue sections were deparaffinized with Xylene and rehydrated with a descending alcohol series. Afterwards, tissue sections were washed in PBS before antigen-retrieval was performed by incubation in a steamer for 20 min in pre-warmed citrate buffer pH 6.0. For all following incubation steps, tissues were placed in a humified chamber. After cool-down to room temperature (RT), endogenous peroxidases were blocked by incubation in stabilized 3% (v/v) H_2_O_2_ solution (Thermo Scientific) for 1 h at RT. Then, quenching of tissue autofluorescence was performed via incubation in 70% (v/v) ethanol in ddH_2_O supplemented with 0.1% (w/v) Sudan Black B (Sigma-Aldrich, Munich, Germany) for 20 min at RT. After washing thrice in PBS, serum block was performed by application of 10% goat serum (Thermo Scientific) on tissue section for 1 h at RT. Hereafter, tissues were incubated over night at 4° C with following primary antibodies: anti-human PD-L1 (clone #E1L3N, 8.76 μg/ml) (Cell Signaling) and anti-human CD68 (clone #514H12, 0.37 μg/ml) (Leica Biosystems, Wetzlar, Germany). Following secondary antibodies were applied for 1 h at RT: goat-anti mouse IgG (H+L)-Alexa Fluor 546 (2 μg/ml) (Thermo Scientific) and goat anti-rabbit IgG-Poly-HRP (provided by the Alexa Fluor 488 Tyramide SuperBoost Kit purchased from Thermo Scientific). Detection of PD-L1 via HRP-antibody complex was achieved by incubation of tissue sections for 10 min in supplied Reaction Cocktail before adding Reaction Stop Solution (both provided by Tyramide SuperBoost Kit, Thermo Scientific). Finally, Hoechst (2 μg/ml) was used for nuclei staining, tissue sections were mounted in FluorSave reagent (Merck Millipore, Darmstadt, Germany) and sealed with clear nail polish by a coverslip. Staining evaluation and image acquisition was performed with a Lionheart FX Automated Microscope (BioTek Instruments, Bad Friedrichshall, Germany).

### Cell lines and cell culture

PDAC cell lines Panc-1, PancTu-I, Panc-89, COLO357, BxPC-3 and were cultured in RPMI 1640 supplemented with 10% fetal calf serum (FCS), 2 mM L-glutamine and 1% sodium pyruvate (all Biochrom, Berlin, Germany). MIAPaCa-2 cells were cultured in DMEM high glucose, 10% FCS, 2.5% horse serum (Thermo Fisher Scientific, Schwerte, Germany) and 1% L-glutamine. Isolation of primary human monocytes from healthy donors and *in vitro* differentiation of macrophages was described previously [42]. Informed consent was obtained from all donors. Authentification of cell lines was performed by STR analysis.

### Western blotting

Preparation of whole-cell lysates, electrophoresis and Western blotting were described previously [48, 49]. The following antibodies were used according to the manufacturer's instructions: rabbit anti-Hsp90α/β (clone H-114, Santa Cruz Biotechnology, Heidelberg, Germany) and rabbit anti-PD-L1 (clone E1L3N).

### Statistical analysis

Statistical analysis was performed using SigmaPlot 12.5 (Systat Software Inc., Chicago, United States of America). All results were examined as raw scores, catagorized by dichotomization and resulting values were compared between groups by Chi-square or Fisher Exact test. Groups of datasets were tested for normal distribution and equal variance by Shapiro-Wilk and Equal Variance test, respectively. Two groups of datasets which failed normality or equal variance test were analyzed by Mann-Whitney Rank Sum test. Non-parametric datasets comprising more than two groups were analyzed by Kruskal-Wallis One-Way Analysis of Variance (ANOVA) on Ranks test. Survival curves were estimated according to Kaplan-Meier method and potential influence factors were identified by Log-Rank test. Statistical significant differences between groups were assumed at p-values < 0.05 and indicated by asterisk (*).

## SUPPLEMENTARY MATERIALS FIGURE AND TABLES



## Abbreviations

α-SMA: α-Smooth muscle actin
CD: Cluster of differentiation
EMT: Epithelial to mesenchymal transition
FoV: Field(s) of view
IHC: Immunohistochemistry
PanCK: Pan Cytokeratin
PanIN: Pancreatic intraepithelial neoplasia
PDAC: Pancreatic ductal adenocarcinoma
PD-L1: Programmed death 1 ligand 1
PD-1: Programmed death protein 1
TAMs: Tumor-associated macrophages
TCR: T cell receptor
