# Supplementary material for: POLE Score: a comprehensive profiling of programmed death 1 ligand 1 expression in pancreatic ductal adenocarcinoma
Source: Oncotarget. 2019 Feb 22;10(16):1572–88. doi: 10.18632/oncotarget.26705 (PMC6422186; doi:10.18632/oncotarget.26705)
Supplement: Supplementary file 1 [file oncotarget-10-1572-s001.pdf]

POLE Score: a comprehensive profiling of programmed death 1 ligand 1 expression in pancreatic ductal adenocarcinoma

SUPPLEMENTARY MATERIALS

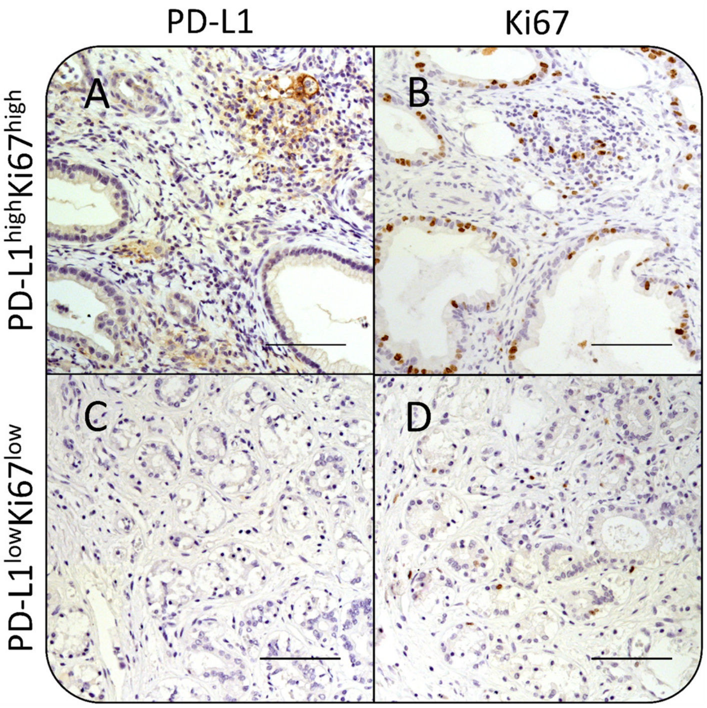

Supplementary Figure 1: Intratumoral areas enriched for PD-L1⁺ stromal cells show high proportion of Ki67⁺ cells within the epithelial tumor compartment. Representative images of (**A**, **C**) PD-L1 and (**B**, **D**) Ki67 immunohistochemical staining in serial pancreatic tissue sections from PDAC patients. Original magnification/scale bar: 200-fold/100 µm.

Supplementary Table 1: Clinic-pathological characteristics of PDAC patients

Parameter	Number of cases
Patients	59
Median age (range)	66 (46–85)
Sex (male/female)	34/25
Tumor stage T1/T2/T3/T4	0/0/58/1
Nodal stage N0/N1	8/51
Metastasis stage M0/M1	57/2
Tumor grade 1 (well differentiated)	7
Tumor grade 2 (moderately differentiated)	27
Tumor grade 3 (poorly differentiated)	24
Survival in months (range)	13 (0–108)

Supplementary Table 2: Statistical correlation of intratumoral PD-L1 expression classified by POLE scoring and stromal as well as epithelial/cancer cell-related parameters of pancreatic tissues of PDAC patients

		PD-L1 expression			
	**Marker**	**+P-Score**	**+O-Score**	**+L-Score**	**E-Score**
**T cells**	CD3+ (% of stroma cells)	0.428	**0.018**	n.p.	0.755
	CD4+ (% of stroma cells)	0.426	0.107	**0.045**	0.193
	CD8+ (% of stroma cells)	1.000	0.107	0.632	0.538
	CD25+ (% of CD3+ cells)	1.000	n.p.	1.000	1.000
	CD25+ (% of CD4+ cells)	1.000	0.464	0.646	0.495
	FoxP3+ (% of CD3+ cells)	0.631	0.464	1.000	1.000
	FoxP3+ (% of CD4+ cells)	1.000	0.464	1.000	1.000
	γδTCR+ (% of stroma cells)	1.000	1.000	0.640	0.492
	γδTCR+ (duct-associated)	1.000	1.000	1.000	0.218
**macrophages**	CD68+ (% of stroma cells)	0.434	1.000	1.000	0.216
	CD68+ (duct-associated)	0.368	n.p.	**0.048**	0.407
	CD163+ (% of stroma cells)	0.447	1.000	1.000	0.230
	CD163+ (duct-associated)	0.368	n.p.	**0.048**	0.407
	HLA-DR+ (% of stroma cells)	0.683	0.429	1.000	1.000
	HLA-DR+ (duct-associated)	0.797	0.446	0.417	0.653
**fibroblasts**	α-SMA+ (% of stroma cells)	1.000	0.429	1.000	1.000
	α-SMA^{high} (α-SMA+)	1.000	**0.036**	0.279	1.000
**EMT**	L1CAM+ (% epithelium)	0.701	0.464	1.000	1.000
	L1CAM+ (intensity epithelium)	0.447	0.464	0.646	1.000
	Vimentin+ (% epithelium)	0.690	1.000	0.621	1.000
**Other**	PanCK+ (% of entire section)	0.338	1.000	0.069	0.524
	Ki67+ (% epithelium)	0.153	n.p.	0.053	1.000
**PD-L1**	+P-Score	/////////	n.p.	0.133	**0.040**
	+O-Score	n.p.	/////////	n.d.	1.000
	+L-Score	0.133	n.d.	/////////	1.000
	E-Score	**0.040**	1.000	1.000	/////////

For statistical analyses POLE Scores were dichotomized (+P, +O, +L, E) into: +P1 (=P0+P1) vs. +P2 (=P2); +O1 (=O1) vs. +O2 (=O2+O3); +L1 (=L0) vs. +L2 (=L1+L2). Results are shown as *p*-values from Chi-square/Fisher exact test. Statistically significant correlations ($p < 0.05$) are marked in bold. n.p. (correlation not possible because one parameter is constant); n.d. (not enough data for statistical analysis).
